# Activation of the alpha 7 nicotinic acetylcholine receptor mitigates osteoarthritis progression by inhibiting NF-κB/NLRP3 inflammasome activation and enhancing autophagy

**DOI:** 10.1371/journal.pone.0256507

**Published:** 2021-12-23

**Authors:** Xianjie Zhu, Shiyou Dai, Baohua Xia, Jianbao Gong, Bingzheng Ma

**Affiliations:** 1 Department of Orthopedics, Qingdao Municipal Hospital, Qingdao, Shandong, China; 2 Department of Clinical Skills Training Center, Qingdao Municipal Hospital, Qingdao, Shandong, China; Chang Gung University, TAIWAN

## Abstract

Osteoarthritis (OA) is a chronic degenerative joint disease characterized by cartilage degradation. Alpha 7 nicotinic acetylcholine receptor (α7nAChR) is associated with inflammatory and metabolic responses in OA. However, the mechanisms underlying the pathological process of OA remain unclear. The aim of the present study was to examine the role and mechanisms of α7nAChR-mediated autophagy and anti-inflammatory response in chondroprotection. Monosodium iodoacetate (MIA)-induced Wistar rat OA model was used to assess the *in vivo* effects of the ɑ7nAChR agonist (PNU-282987). The histopathological characteristics of OA were evaluated by immunohistochemistry (IHC), and the levels of autophagy markers were determined by western blotting and transmission electron microscopy. The anti-inflammatory effect of the ɑ7nAChR agonist was assessed by IHC, quantitative real-time polymerase chain reaction, and western blotting. Parallel experiments to determine the molecular mechanisms through which the ɑ7nAChR agonist prevents OA were performed using interleukin-1β (IL-1β)-treated chondrocytes. Our results showed that PNU-282987 reduced cartilage degeneration and matrix metalloproteinase (MMP)-1 and MMP-13 expressions. Activating α7nAChR with PNU-282987 significantly promoted MIA/IL-1β-induced chondrocyte autophagy, as demonstrated by the increase in LC3-II/LC3-I ratio, Beclin-1 levels, and autophagosome number. Furthermore, treating chondrocyte with ULK1 siRNA attenuated the PNU282987-induced enhancement of LC3-II/LC3-I ratio and Beclin-1 level. Additionally, PNU282987 suppressed NF-κB/NLRP3 inflammasome activation by inhibiting the ROS/TXNIP pathway and suppressed tumor necrosis factor-ɑ and IL-1β secretion in MIA/IL-1β-treated chondrocytes. Our results demonstrate that the activation of α7nAChR promotes chondrocyte autophagy and attenuates inflammation to mitigate OA progression, providing a novel target for the treatment of OA.

## Introduction

Osteoarthritis (OA) is a highly prevalent disease and a leading cause of disability and chronic pain [1, 2]. OA is characterized by the progressive breakdown of articular cartilage and remodeling of the synovial joints, predominantly affecting the knees, hips, spine, and fingers. The personal and social burden of OA is increasing, and the current treatment options lack disease mitigation abilities and are limited to pain relief to maintain joint function. Ultimately, the only definitive treatment option is surgical joint replacement. Thus, safe and effective therapeutic drugs for the early treatment of OA must be explored.

Nicotinic acetylcholine receptors (nAChRs) are composed of five subunits: ɑ(1–10), β(1–4), γ, δ, and ε. In particular, the α7 (α7nAChR) subtype is important for immune regulation [3]. Researchers have previously demonstrated that the activation of α7nAChR can help reduce a variety of inflammatory and immune-related diseases by triggering the cholinergic anti-inflammatory pathway [4, 5]. Recent evidence indicates that α7nAChR has a relieving effect on OA [6]. However, the underlying mechanisms by which α7nAChR functions have not been fully elucidated.

Autophagy is a highly conserved process of lysosome-mediated degradation of long-lived proteins and damaged organelles to maintain cellular homeostasis and metabolism [7]. It has been reported that inducing autophagy can ameliorate several degenerative diseases [8]. Autophagy not only regulates the last stage of the chondrocyte life cycle, but also regulates the rate at which chondrocytes enter the maturation process [9]. Recent studies have shown that the level of autophagy in osteoarthritic cartilage is reduced and that autophagy can protect chondrocytes from degradation [10]. However, whether autophagy is involved in a α7nAChR-mediated process in OA remains unclear.

Many studies have confirmed that activated nuclear factor-kappa B (NF-κB) can induce the overexpression of a variety of matrix metalloproteases (MMPs), leading to the occurrence and development of OA [11]. Chondrocytes and synovial cells in OA produce or overproduce various inflammatory mediators, such as interleukin-1β (IL-1β), tumor necrosis factor-alpha (TNF-ɑ), and nitric oxide (NO), which are characteristic of inflammatory arthritis [12]. The NOD-, LRR- and pyrin domain-containing protein 3 (NLRP3) inflammasome, composed of NLRP3, an apoptosis-related speckle-like protein-containing acupaspase recruitment domain (ASC) and pro-caspase-1, is the most studied member of the Nod-like receptor (NLR) family [13]. In a recent study, the expression levels of NLRP3 in the synovial membrane of patients with knee OA increased by 5.4 times compared with that of the control group [14]. This result emphasizes the potential role of NLRP3 in OA and the possibility of its measurement as a biomarker for OA or its targeted inhibition.

In this study, we hypothesized that α7nAChR-alleviated OA occurs via the induction of autophagy and mitigation of inflammation in chondrocytes. We aimed to explore the pathogenesis of OA and provide new insights into the development of new strategies for the treatment of OA.

## Materials and methods

### Animals

Specific pathogen-free Wistar rats (male, 280–320 g, 3-months old) were purchased from Hunan SJA Laboratory Animal Co., Ltd. (Changsha, China). The rats were kept under standard laboratory conditions (24°C; 12-h light-dark cycles). All animal experiments were conducted according to the Guiding Principles in the Care and Use of Laboratory Animals published by the U.S. National Institutes of Health (NIH Publication No. 8023, revised 1978) and were approved by the Animal Ethics Committee of Qingdao University. After one week of acclimatization, the OA model was prepared according to a previous study [15]. Briefly, for establishment of MIA-induced arthritis, 1 mg of monosodium iodoacetate (MIA; Sigma-Aldrich, St. Louis, MO, USA) in 50 μL of sterile physiologic saline solution was injected into right knee joints through the infrapatellar ligament. Control group were treated with saline. Two weeks after injection with MIA, rats were given intraperitoneal injections of PNU-282987 (4.8 mg/kg in citrate buffer) once per day for 45 days. Forty five days after injection of PNU-282987, the rats were sacrificed and the specimens of the knee joint were collected for the follow-up experiment.

### Histopathology and Immunohistochemistry (IHC)

Cartilage blocks were immersed in 10% neutral buffered formalin at 4°C for three days, followed by decalcification for 14 days in 30% formic acid solution and dehydration with ethanol in a conventional gradient. The sample was embedded in paraffin and cut into 5-μm sections.

For hematoxylin and eosin (H&E) staining, the paraffin sections were dewaxed, hydrated with graded ethanol, stained with hematoxylin solution for 15 min, and counterstained with eosin solution for 5 min. After dehydration, transparency induction, and sealing with gradient alcohol, the pathological condition of the articular cartilage was observed using Image-Pro image analysis software.

For Safranin O/Fast green staining, the samples were stained with 0.5% Fast Green for 20 min and then 0.5% Safranin O for 5 min, followed by dehydration with gradient alcohol, transparency induction with xylene, and sealing with neutral gum. Normal cartilage appeared red, and the background appeared green.

Immunohistochemical staining was performed according to the manufacturer’s instructions (Solarbio, Beijing, China), and samples were observed under a microscope (Olympus, Tokyo, Japan).

### ELISA

According to the manufacturer’s instructions, supernatants of rat sera were used to measure IL-1β and TNF-ɑ levels (R&D Systems, Minneapolis, MN, USA).

### Isolation and culture of chondrocytes

Cartilage sections were shaved from the joint surfaces of the knee joints of adult male Wistar rats. All rats were killed by spinal cord dislocation finally. Cartilage samples were digested in 0.25% trypsin at 37°C for 1 h and then transferred to 0.3% collagenase II at 37°C for 6 h until the extracellular matrix was completely digested. Chondrocytes were filtered through a mesh, and the resulting single-cell suspension was centrifuged at 1500 × g for 10 min. Then, the cells were transferred to a culture flask and incubated with complete Dulbecco’s modified Eagle’s medium (DMEM) in 5% CO_2_ at 37°C. Chondrocytes were identified using collagen II immunohistochemical staining.

Target cells were grouped as follows: for IL-1β-induced injury model in chondrocytes, chondrocytes were stimulated with 10 ng/mL IL-1β (Shanghai Sangon Biotech Co., Ltd., China) for 24 h; for IL-1β and PNU-282987 combination, chondrocytes were preprocessed with 10 μm PNU-282987 for 12 h and followed by co-processing with 10 ng/mL IL-1β for 24 h.

### Cell viability

Rat chondrocytes were seeded in 96-well plates (1.0 × 10^3^–3.0 × 10^3^/well) and treated with PNU-282987 (10μM) for 24, 48, and 72 h. After adding 20 mL of 3-(4,5-Dimethylthiazol-2-yl)-2,5-diphenyltetrazolium bromide (MTT) solution (5 mg/mL) (Sigma-Aldrich, St. Louis, MO, USA) to each well, the plate was incubated at 37°C in 5% CO2 for 4 h. After removing the supernatant and dissolving the cells in dimethyl sulfoxide (DMSO; 150 μL/well), the absorbance was measured at 570 nm using a microplate reader (Leica, Wetzlar, Germany).

### Transmission electron microscopy (TEM)

Rat chondrocytes were fixed with 2.5% glutaraldehyde, dehydrated with graded ethanol, and embedded in epoxy resin. Ultrathin sections were observed using a transmission electron microscope (JEM-1230; JEOL, Tokyo, Japan).

### Small interfering RNA (siRNA) transient transfection

NLRP3 and ULK1 siRNA targeting rat chondrocytes were obtained from GenePharma (Shanghai, China). siRNA (100 nM) transfection was performed using Lipofectamine RNAiMax (Invitrogen, Carlsbad, CA, USA) according to the manufacturer’s instructions. The target sequence of the ULK1 siRNA was 5- GGUACCACCAGAGCAACAUTT-3′; the target sequence of the NLRP3 siRNA was 5-GGTGTTGGAATTAGACAAC-3′. Cells were subjected to IL-1β post-transfection.

### Flow cytometric analysis

A peroxide-sensitive fluorescent probe 2’,7’-dichlorofluorescein diacetate (DCFH-DA) was used to detect reactive oxygen species (ROS) levels. After treatment, chondrocytes were washed three times with phosphate buffered saline (PBS) and incubated with DCFH-DA for 30 min in the dark. Fluorescence was detected using a flow cytometer (BD Biosciences, San Jose, CA, USA).

### RNA extraction and quantitative real-time polymerase chain reaction (RT-qPCR)

After incubation, total RNA was extracted from chondrocytes and cartilage tissue using TRIzol (Invitrogen, Carlsbad, CA, USA) according to the manufacturer’s instructions and **was** reverse-transcribed to cDNA with RT Master Mix (Takara Bio, Kyoto, Japan). RT-qPCR was performed with a 7300 Real-Time PCR System using SYBR Green PCR Master Mix. Denaturation was done at 95°C for 30 s, annealing at 60°C for 1 min, and extension at 95°C for 5 s. The glyceraldehyde-3-phosphate dehydrogenase (GAPDH) fragment was amplified as an internal control. The primer sequences were as follows: NLRP3 forward (5’-GTAGGTGTGGAAGCAGGACT-3’) and reverse (5’-CTTGCTGACTGAGGACCTGA-3′), ASC forward (5’-AGTTTCACACCAGCCTGGAA-3’) and reverse (5’-TTTTCAAGCTGGCTTTTCGT-3′), caspase-1 forward (5’-CCGAAGGTGATCATCATCCA-3′) and reverse (5’-ATAGCATCATCCTCAAACTCTTCTG-3′), TXNIP forward (5’-GCTCAATCATGGTGATGTTCAAG-3’) and reverse (5’-CTTCACACCACTTCCACTGTCAC-3′), and GAPDH forward (5′-CAAGTTCAACGGCACAG-3nc) and reverse (5′-CCAGTAGACTC CACGACAT-3′).

### Western blot analysis

Cartilage tissue and chondrocytes were homogenized in a lysate containing protease inhibitors and protein extraction was performed as previously described [16].

Western blotting was performed according to the manufacturer’s instructions. The primary antibodies used were as follows: NLRP3, ASC, thioredoxin-interacting protein (TXNIP), and caspase-1 (1:1000, Abcam, Cambridge, MA, USA); MMP-1, MMP-13, collagen II, and Unc-51-like kinase 1 (ULK1) (1:2000, Abcam, Cambridge, MA, USA);p-NF-κB and inhibitor kappa B-alpha (IκB-ɑ) (1:500, Cell Signaling Technology, Danvers, MA, USA); and Beclin-1, light chain 3 (LC3), GAPDH, and β-actin (1:500–1:2000, Santa Cruz Biotechnology, Santa Cruz, CA, USA). Antigen-antibody complexes were visualized using an ECL Western Detection Kit (Thermo Fisher Scientific, Waltham, MA, USA). Protein levels were normalized to the levels of β-actin or GAPDH. ImageJ software was used to quantify the density of each band.

### Statistical analysis

Data are expressed as the mean ± standard deviation (SD). Statistical significance was analyzed by Student’s t-test for two groups or one-way analysis of variance (ANOVA) followed by Tukey’s post-hoc test for multiple comparisons using GraphPad Prism version 6.0. Results were considered significant at P < 0.05.

## Results

### Activation of a7nAChR alleviates histopathological osteoarthritic changes and promotes autophagy in an OA rat model

**Fig 1:**
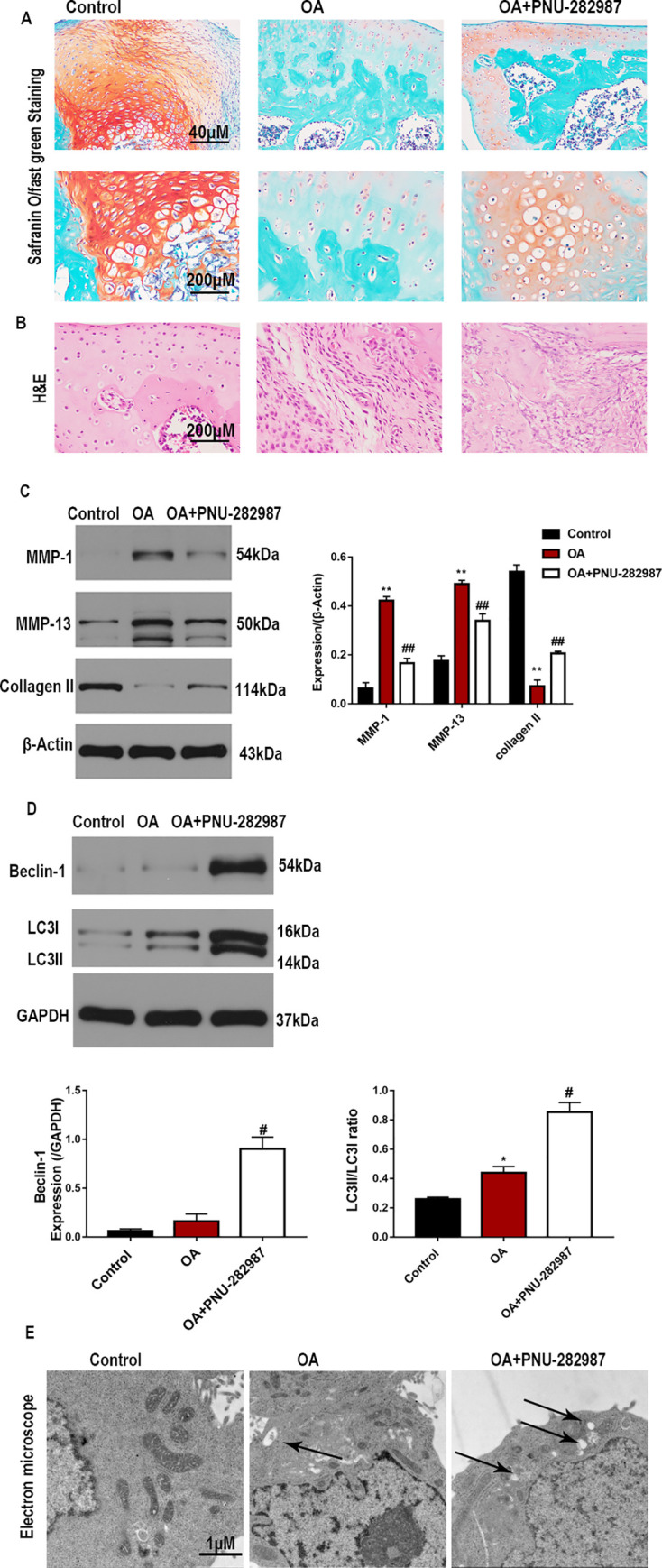
α7nAChR activation protects against cartilage injury and promotes autophagy in rats with OA. (A) Safranin O/Fast Green staining and (B) H&E staining were used to evaluate cartilage histopathology in rats with OA. (C) Expressions of MMP-1, collagen II, and MMP-13 were evaluated by western blotting and quantification analyses. (D) Western blotting was used to detect the protein levels of Beclin-1 and LC3, and (E) TEM was used to analyze the autophagosome number. n = 5–10 per group, *P < 0.05, **P < 0.01 vs. Control group; #P < 0.05, ##P < 0.01 vs. OA group.

The effect of the ɑ7nAChR agonist on the histological morphological structure of tissues from the OA group was evaluated. The results of H&E and Safranin O/Fast Green staining showed more severe cartilage erosion in the rat OA group than in the control group. Treatment with PNU-282987 alleviated the degeneration and erosion of articular cartilage (Fig 1A and 1B). We then assessed the effects of the ɑ7nAChR agonist on the accumulation of OA markers by western blotting. The results revealed that the protein levels of MMP-1 and MMP-13 were suppressed, while the protein level of collagen II was increased after PNU-282987 treatment *in vivo* (Fig 1C).

Autophagy dysfunction is known to be involved in OA. To examine the effects of a7nAChR activation on autophagy in the OA model, we measured the protein levels of LC3 and Beclin-1. As shown in Fig 1D, the LC3II/LC3-I ratio and Beclin-1 levels were increased in the OA group, indicating stimulated autophagy, and PNU-282987 treatment further increased the levels of autophagy-related proteins. Furthermore, TEM revealed few autophagosomes in the OA group, and treatment with the ɑ7nAChR agonist increased the number of intracellular autophagosomes (Fig 1E). These results demonstrate that the activation of a7nAChR can reduce articular cartilage damage and promote protective autophagy in rats with OA.

### Activation of a7nAChR inhibits NF-κB/NLRP3 inflammasome activation

**Fig 2:**
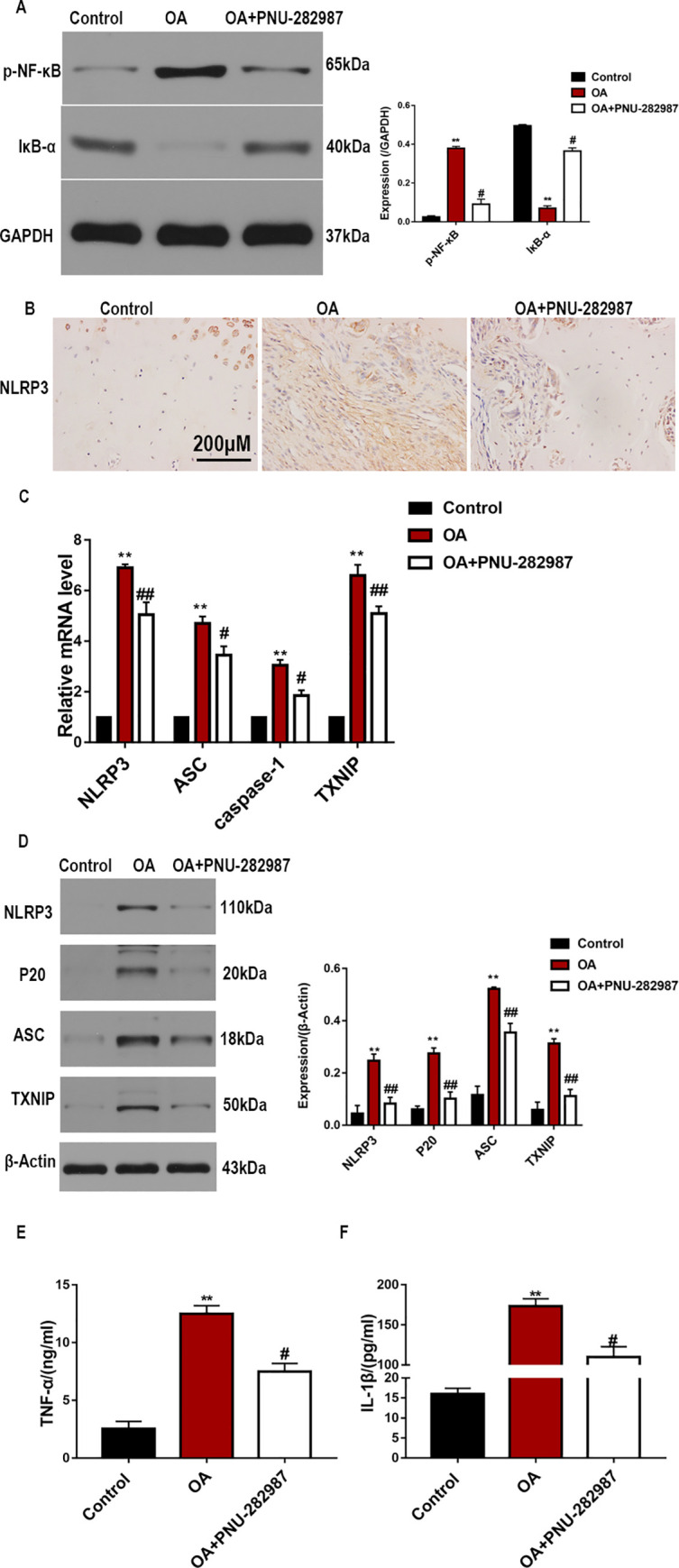
α7nAChR activation suppresses NF-κB/NLRP3 inflammasome activation in rats with OA. (A) IκB-α and p-NF-κB protein levels were evaluated by western blotting. (B) Representative immunohistochemical staining for NLRP3 expression in rats with OA. (C) mRNA levels of NLRP3, ASC, caspase-1, and TXNIP in joint tissues were evaluated by RT-qPCR. (D) Protein levels of NLRP3, ASC, cleaved-caspase-1 (p20), and TXNIP in joint tissues were evaluated by western blotting. ELISA results showing serum levels of TNF-ɑ (E) and IL-1β (F) in the study groups. n = 5–10 per group, *P < 0.05, **P < 0.01 vs. Control group; #P < 0.05, ##P < 0.01 vs. OA group.

The transcription factor NF-κB is closely related to inflammatory reactions and matrix metabolism during OA progression. We analyzed the effect of the ɑ7nAChR agonist on the NF-κB pathway in cartilage tissue. As shown in Fig 2A, OA increased the protein level of p-NF-κB, which is related to the decrease in the level of IκB-α. However, PNU-282987 significantly inhibited the degradation of IκB-α and reversed the expression of p-NF-κB. To further investigate the effect of the ɑ7nAChR agonist on chondrocyte inflammation in the OA model, IHC was performed to detect the level of NLRP3. As shown in Fig 2B, the rat OA model exhibited a marked increase in NLRP3 levels; however, PNU-282987 caused a marked reduction in the number of positive cells in the rat cartilage tissue. The expression of the NLRP3 inflammasome in joint tissues was analyzed by RT-qPCR and western blotting. The results, which showed that the ɑ7nAChR agonist downregulated the gene expressions of NLRP3, ASC, TXNIP, and caspase-1 in rats with OA (Fig 2C), was consistent with the results of the protein analysis (Fig 2D). In addition, PNU-282987 reduced the levels of mature IL-1β and TNF-α in sera of rats with OA (Fig 2E and 2F). Overall, these results indicate that PNU-282987 exerts a protective effect against OA by regulating the activation of the NF-κB/NLRP3 pathway.

### Activation of a7nAChR regulates autophagy through ULK1 in chondrocytes

**Fig 3:**
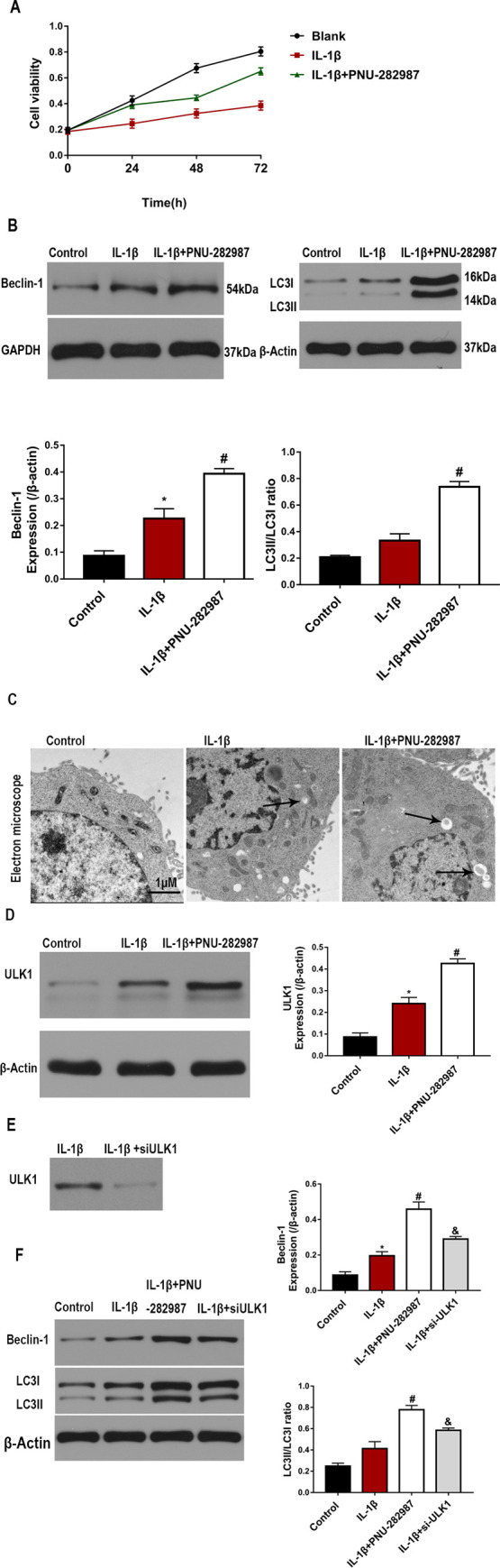
α7nAChR activation promotes autophagy in IL-1β-treated chondrocytes. (A) Proliferation rate of IL-1β-treated chondrocytes was measured via MTT assay at 24, 48, and 72 h after transfection. (B) Expressions of LC3 and Beclin-1 in chondrocytes were evaluated by western blotting. (C) Representative TEM images of autophagosomes in chondrocytes (arrows indicate intracellular autophagosomes). Scale bars: 1 μm. (D) Expression of ULK1 in chondrocytes was evaluated by western blotting. (E) Chondrocytes were pre-treated with PNU-282987 or ULK1 siRNA for 2 h and then incubated with IL-1β. (F) Relative protein levels of LC3 and Beclin-1 in chondrocytes were evaluated by western blotting. n = 3, *P < 0.05, **P < 0.01 vs. Control group; #P < 0.05, ##P < 0.01 vs. IL-1β group; &P < 0.05, &&P < 0.01 vs. IL-1β+ PNU-282987 group.

To analyze the effects of the ɑ7nAChR agonist on the proliferation and viability of IL-1β-treated chondrocytes, we pretreated the cells with PNU-282987. MTT assay showed that at 24, 48, and 72 h after IL-1β stimulation, the growth rate of chondrocytes was significantly inhibited, but was completely restored after pretreatment with the ɑ7nAChR agonist (Fig 3A).

To further validate the potential effect of the ɑ7nAChR agonist on autophagy, we performed an *in vitro* assay. The results showed that IL-1β increased the LC3-II/LC3-I ratio and Beclin-1 levels, and PNU-282987 further increased the levels of autophagy-related proteins (Fig 3B). Furthermore, TEM analysis validated the increase in the number of intracellular autophagosomes in PNU-282987-treated chondrocytes (Fig 3C). To further clarify the underlying mechanisms of ɑ7nAChR agonist-induced autophagy in damaged chondrocytes, we analyzed the levels of ULK1 in IL-1β-treated cells. ULK1 levels were increased in the IL-1β group, and treatment with the ɑ7nAChR agonist further augmented the expression of ULK1 in response to IL-1β (Fig 3D). Therefore, we hypothesised that the ULK1 signalling may in part be responsible for controlling autophagy regulation of ɑ7nAChR.We transfected chondrocytes with ULK1 siRNA(Fig 3E). Indeed, silencing of ULK1 in PNU-282987 treated chondrocytes resulted in a significant decrease in the expression of LC3II /LC3I as well as expression of Beclin-1(Fig 3F).

### Activation of a7nAChR suppresses IL-1β-mediated NF-κB/NLRP3 inflammasome activation in chondrocytes

**Fig 4:**
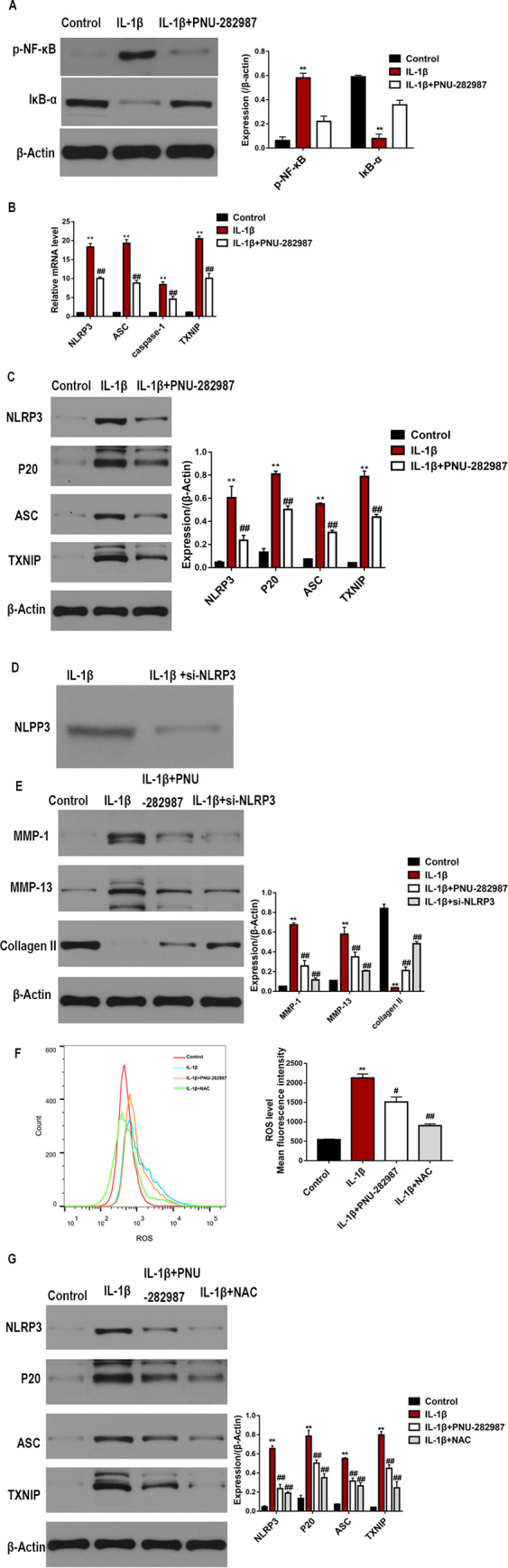
α7nAChR activation inhibits NF-κB/NLRP3 inflammasome activation in IL-1β-treated chondrocytes. (A) Protein levels IκB-α and p-NF-κB were evaluated by western blotting. (B) mRNA levels of NLRP3, ASC, caspase-1, and TXNIP in chondrocytes were detected by RT-qPCR and normalized to those of GAPDH. (C) Relative protein levels of NLRP3, ASC, cleaved-caspase-1 (p20), and TXNIP in lysates of chondrocytes were determined by western blotting. Chondrocytes were transfected with NLRP3 siRNA. (D) Western blot analysis revealed the successful knockdown of NLRP3. (E) Chondrocytes were pre-treated with PNU-282987 or NLRP3 siRNA for 2 h and then incubated with IL-1β. Relative protein levels of MMP-1, Collagen II, and MMP-13 in lysates of chondrocytes were determined by western blotting and normalized to those of β-actin. (F) Flow cytometric histogram of chondrocytes in different groups and quantification analyses. (G) Chondrocytes were pre-treated with PNU-282987 or NAC for 2 h and then incubated with IL-1β. Expressions of NLRP3, ASC, cleaved-caspase-1 (p20), and TXNIP in chondrocytes were evaluated by western blotting. n = 3, *P < 0.05, **P < 0.01 vs. Control group; #P < 0.05, ##P < 0.01 vs. IL-1β group.

Then, we determined whether the ɑ7nAChR agonist can inhibit NF-κB activation in chondrocytes. In chondrocytes, IL-1β increases p-NF-κB levels and reduces IκB-α levels. Pretreatment with the ɑ7nAChR agonist reduced the activation of NF-κB (Fig 4A). Moreover, the RT-qPCR and western blotting results indicated that the ɑ7nAChR agonist reversed the IL-1β-induced increase in the expressions of NLRP3, ASC, and caspase-1 (Fig 4B and 4C). NLRP3 siRNA was used to further investigate the effect of the ɑ7nAChR agonist on IL-1β-treated chondrocytes. Western blotting confirmed the successful transfection (Fig 4D). As shown in Fig 4E, NLRP3 knockdown resulted in the significant downregulation of MMP-1 and MMP-13 expressions, but reversed the downregulation of collagen II expression in IL-1β-treated chondrocytes. This effect was comparable to that induced by the ɑ7nAChR agonist. Furthermore, to investigate the regulatory mechanisms underlying these effects, we examined the generation of ROS. Chondrocytes were pre-treated with the ɑ7nAChR agonist or N-acetyl cysteine (NAC) and then stimulated with IL-1β. The results showed that the ɑ7nAChR agonist inhibited the IL-1β-induced generation of ROS, and this effect was comparable to that induced by NAC (Fig 4F). In addition, we found that NAC reduced TXNIP and NLRP3 inflammasome expressions (Fig 4G). The above findings indicate that the activation of ɑ7nAChR suppresses the NF-κB/NLRP3 signaling pathway, thereby influencing inflammatory processes.

## Discussion

There are two major findings in this study. First, we demonstrated that the activation of α7nAChR can restore ULK1expression and counter chondrocyte autophagy. Second, we provided direct evidence that the activation of α7nAChR attenuates chondrocyte injury and inflammatory response by suppressing NF-κB/NLRP3 inflammasome activation. These results demonstrate a newly explored mechanism involving α7nAChR-associated chondroprotection in OA, providing a potential therapeutic target to prevent or slow OA progression.

Several studies have reported that activating α7nAChR alleviates cartilage injury in OA. In particular, Gu et al. demonstrated that α7nAChR activation attenuated cellular damage, increased ECM synthesis, and reduced serum TNF-α levels in a rat model of early-stage OA [17]. Furthermore, Teng et al. [18] reported that the stimulation of α7nAChR by nicotine attenuated MIA-induced OA pain and cartilage degradation and that the protective effect of nicotine can be associated with the inhibition of MMP-9 overexpression through the phosphoinositide-3-kinase (PI3K)/Akt/NF-κB signaling pathway. Consistent with these previous studies, our study showed that activating α7nAChR using PUN-282987 considerably reduced the severity of OA-induced cartilage injury *in vitro* and *in vivo*.

It is of interest to explore the possible mechanisms by which α7nAChR protects against OA. It has been previously reported that the activation of α7nAChR plays an active role in the fight against diseases by promoting autophagy in several cells [19]. Jeong et al. recently demonstrated that the activation of α7nAChR contributed to the induction of neuronal autophagic flux, which plays a key role in neuroprotection [20]. Shao et al. demonstrated that autophagy alleviated the effects of inflammatory bowel disease by activating α7nAChR and that α7nAChR knockout greatly inhibited autophagy in the colon [21]. In the current study, we used PNU-282987 to selectively activate α7nAChR, which significantly increased the levels of autophagy-related proteins in chondrocytes and regulated the autophagy dysfunction in rat cartilage. It has been previously reported that mammalian target of rapamycin (mTOR)/ULK1 signaling pathway serves as a classic pathway in the induction of autophagy in OA [22] and that targeting downstream autophagy-related proteins by binding to and activating ULK1 can protect against OA, alluding to a separate process of autophagy regulation independent of the mTOR/ULK1 signaling pathway [23]. We found that ULK1 is involved in α7nAChR-mediated changes in autophagy markers in chondrocytes.

α7nAChR is considered the most important receptor for transmitting cholinergic anti-inflammatory signals. The activation of NLRP3 inflammasome is mediated at transcriptional and post-translational levels by the toll-like receptor (TLR)/ NF-κB pathway [24]. The priming step activates the inflammatory process in cells and upregulates the expression of inflammasome components upon increased transcriptional activity of NF-Κb [25].

Genes encoding inflammatory proteins are upregulated during OA, primarily through signal transduction involving NF-κB, and other inflammation- and stress-induced pathways [26].

Activation of the NF-κB/NLRP3 inflammasome upregulates the production of IL-1β and TNF-ɑ, which are the main cartilage-degrading cytokines in OA [27]. A recent study reported that the activation of α7nAChR inhibited the NLRP3 inflammasome, helping control neuroinflammation in mice with autoimmune encephalomyelitis [28]. Moreover, a study by Jiang et al. emphasized that the upregulation of α7nAChR expression in neurons inhibited NLRP3 inflammasome-related inflammatory response, reduced apoptosis, and regulated the balance between pro-inflammatory factors and anti-inflammatory cytokines after transient cerebral ischemia [29]. However, the underlying mechanisms involving α7nAChR and the NLRP3 inflammasome remain to be explored. ROS generation may be involved in these mechanisms, as ROS can activate the NLRP3 inflammasome, and activated α7nAChR can inhibit oxidative stress during inflammation [30, 31]. As expected in this study, activated α7nAChR inhibited the activation of the NF-κB/NLRP3 inflammasome and the release of inflammatory factors, such as IL-1β, both *in vitro* and *in vivo*. Our *in vitro* experiments showed that the ɑ7nAChR agonist and ROS inhibitors significantly reduced ROS production and inhibited NLRP3 inflammasome activation.

In this study, we demonstrated that the activation of α7nAChR can mitigate OA progression through NF-κB/NLRP3 inflammasome inhibition and ULK1-mediated autophagy, revealing potential and promising therapeutic strategies for the treatment of OA. However, the interaction between autophagy and the NLRP3 inflammasome in the pathogenesis of OA requires further investigation.

## Supporting information

S1 Raw images (PDF)

## Abbreviations

OA: osteoarthritis
nAChR: nicotinic acetylcholine receptor
α7nAChR: alpha 7 nicotinic acetylcholine receptor
NF-κB: nuclear factor-kappa B
MMP: matrix metalloprotease
IL-1β: interleukin 1β
TNF-ɑ: tumor necrosis factor-alpha
NO: nitric oxide
NLRP3: NOD-, LRR- and pyrin domain-containing protein 3
ASC: acupaspase recruitment domain
NLR: Nod-like receptor
MIA: monosodium iodoacetate
IHC: Immunohistochemistry
DMEM: Dulbecco’s modified Eagle’s medium
MTT: 3-(4,5-Dimethylthiazol-2-yl)-2,5-diphenyltetrazolium bromide
DMSO: dimethyl sulfoxide
TEM: Transmission electron microscopy
siRNA: small interfering RNA
DCFH-DA: 2’,7’-dichlorofluorescein diacetate
ROS: reactive oxygen species
PBS: phosphate buffered saline
RT-qPCR: quantitative real-time polymerase chain reaction
GADPH: glyceraldehyde-3-phosphate dehydrogenase
TXNIP: thioredoxin-interacting protein
IκB-ɑ: inhibitor kappa B-alpha
SD: standard deviation
ANOVA: one-way analysis of variance
NAC: N-acetyl cysteine
PI3K: phosphoinositide-3-kinase
mTOR: mammalian target of rapamycin
