# Supplementary material for: Activation of the alpha 7 nicotinic acetylcholine receptor mitigates osteoarthritis progression by inhibiting NF-κB/NLRP3 inflammasome activation and enhancing autophagy
Source: PLoS One. 2021 Dec 23;16(12):e0256507. doi: 10.1371/journal.pone.0256507 (PMC8699641; doi:10.1371/journal.pone.0256507)

Fig.1C (rat cartilage tissue)
(control OA OA+PNU-282987)

MMP-1(54kDa)

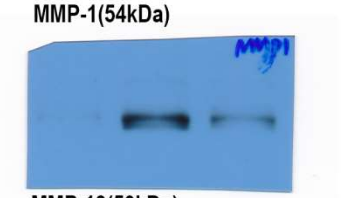

MMP-13(50kDa)

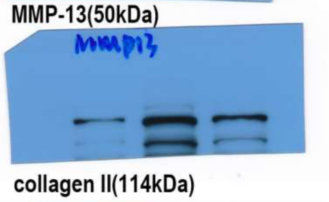

collagen II(114kDa)

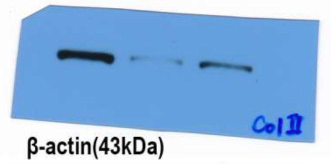

β-actin(43kDa)

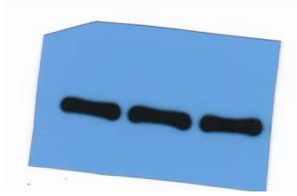

Fig.1D(rat cartilage tissue)
(control OA OA+PNU-282987)

Beclin-1(54kDa)

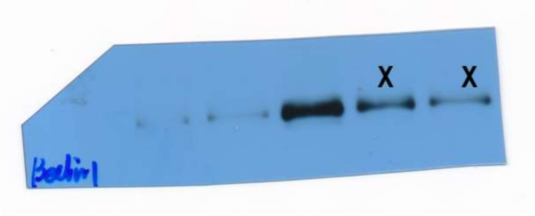

LC3II(14kDa) LC3I(16kDa)

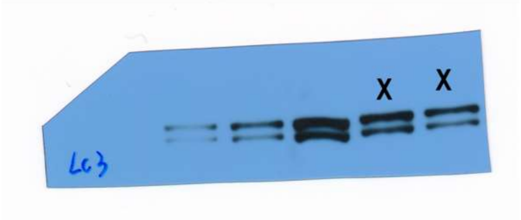

GAPDH(37kDa)

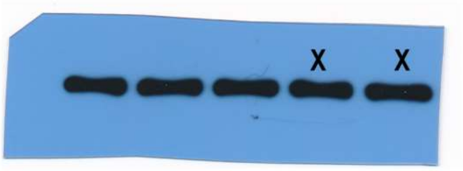

Fig.2A(rat cartilage tissue)
(control OA OA+PNU-282987)

p-NF-κB(65kDa)

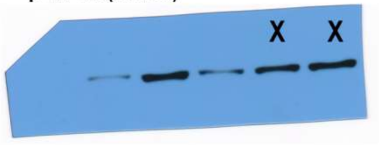

IκB-α(40kDa)

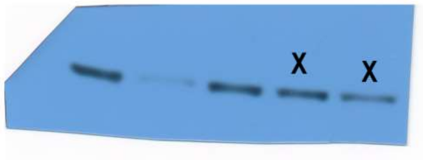

GAPDH(37kDa)

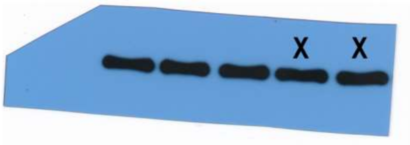

Fig.2D(rat cartilage tissue)
(control OA OA+PNU-282987)

NLRP3(110kDa)

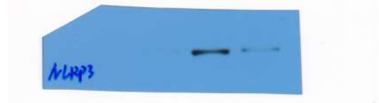

P20(20kDa)

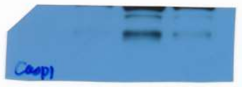

ASC(18kDa)

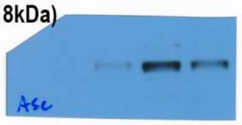

TXNIP(50kDa)

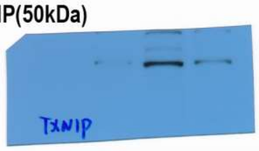

β-actin(43kDa)

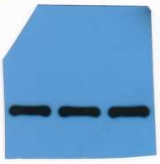

Fig.3B (chondrocytes)

(Control IL-1β IL-1β+PNU-282987)

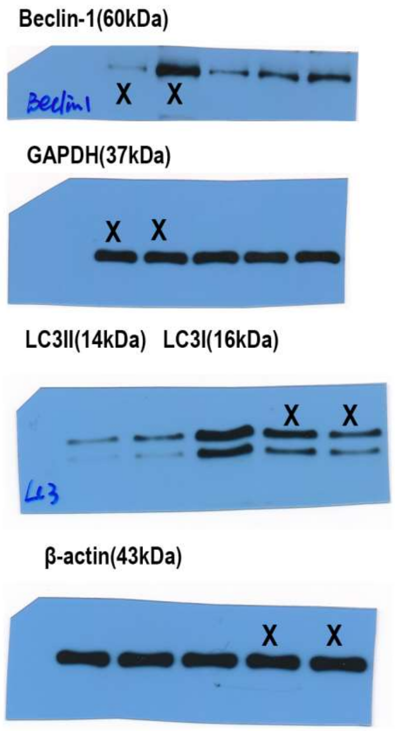

Fig.3D(chondrocytes)

(Control IL-1β IL-1β+PNU-282987)

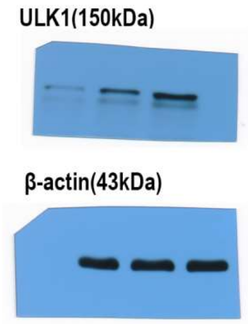

Fig.3E(chondrocytes)

(IL-1β IL-1β+si-ULK1)

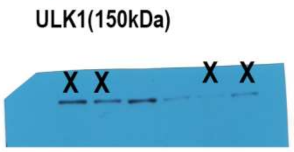

Fig.3F(chondrocytes)

(Control IL-1β IL-1β+PNU-282987

IL-1β+si-ULK1)

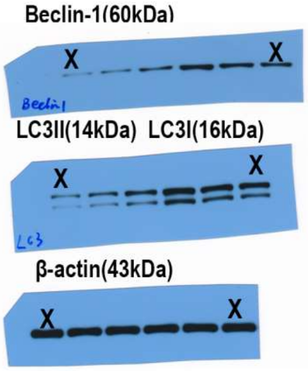

Fig.4A (chondrocytes)

(Control IL-1β IL-1β+PNU-282987)

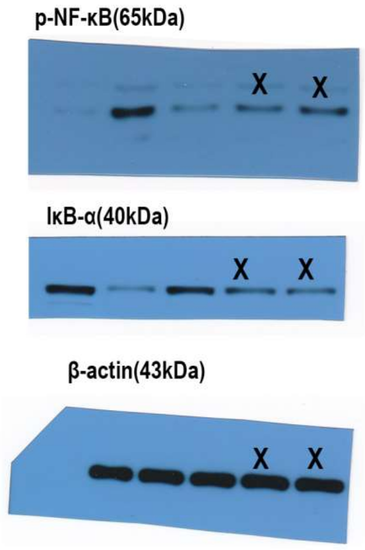

Fig.4C (chondrocytes)

(Control IL-1β IL-1β+PNU-282987)

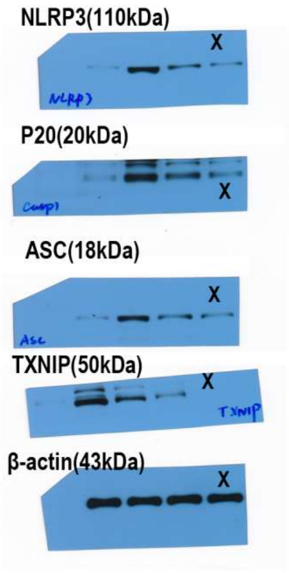

Fig.4D (chondrocytes)

(IL-1β IL-1β+si-NLRP3)

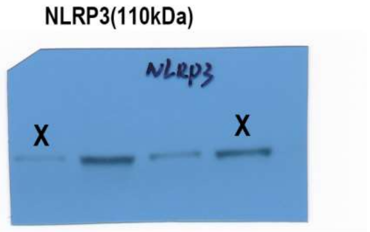

Fig.4E (chondrocytes)

(Control IL-1β IL-1β+PNU-282987 IL-1β+si-NLRP3)

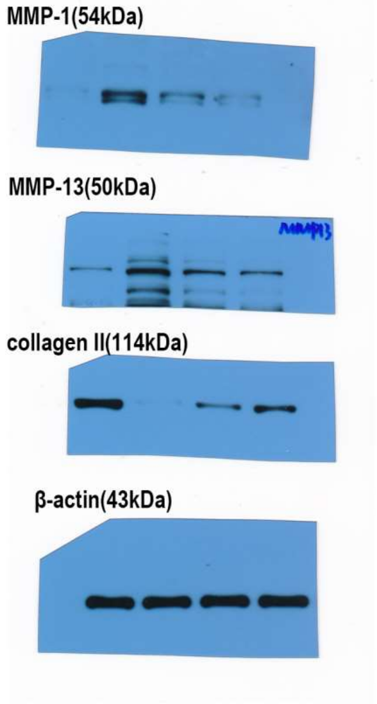

Fig.4G (chondrocytes)

(Control IL-1β IL-1β+PNU-282987 IL-1β+NAC)

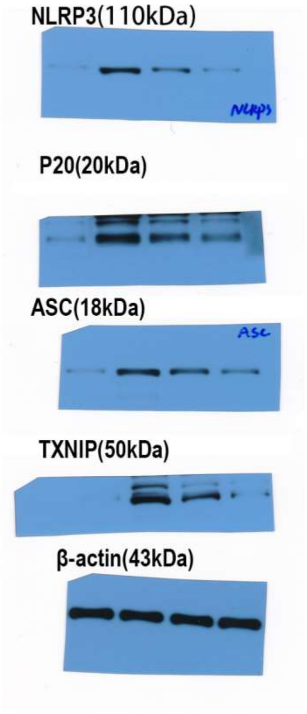

Supplement: S1 Raw images — (PDF) [file pone.0256507.s001.pdf]
